# Iodine-123-meta-iodobenzylguanidine Myocardial Scintigraphy in Isolated Autonomic Failure: Potential Red Flag for Future Multiple System Atrophy

**DOI:** 10.3389/fneur.2017.00225

**Published:** 2017-05-26

**Authors:** Francesca Baschieri, Giovanna Calandra-Buonaura, Annagrazia Cecere, Giorgio Barletta, Manuela Contin, Piero Parchi, Pietro Cortelli

**Affiliations:** ^1^Clinica Neurologica, Dipartimento di Medicina, Università degli Studi di Perugia, Ospedale S. Maria della Misericordia, Perugia, Italy; ^2^Department of Biomedical and Neuromotor Sciences, University of Bologna, Bologna, Italy; ^3^IRCCS, Institute of Neurological Sciences, Bellaria Hospital, Bologna, Italy

**Keywords:** autonomic failure, Parkinson’s disease, multiple system atrophy, Iodine-123-meta-iodobenzylguanidine myocardial scintigraphy, orthostatic hypotension

## Abstract

Pure autonomic failure is challenging as it can be the presenting feature of a central nervous system syncleinopathy such as Parkinson’s disease (PD) or multiple system atrophy (MSA). Because the prognosis of MSA and PD is so different, predictive features for a possible conversion can be extremely valuable. In this paper, we report three cases (two with autopsy-proven diagnosis) that had isolated AF for many years before converting to MSA or PD. Of all the tests that were performed during the premotor stage, Iodine-123-meta-iodobenzylguanidine (MIBG) myocardial scintigraphy was predictive of the conversion to MSA. We suggest that MIBG myocardial scintigraphy, when performed in patients with isolated AF, may be a valuable predictor of conversion to MSA. On the contrary, the role of such test in parkinsonian patients irrespective of the presence of AF is still to be clarified.

## Introduction

Pure autonomic failure (PAF) is defined as an idiopathic sporadic disorder characterized by orthostatic hypotension (OH) usually with evidence of more widespread AF and no other neurological signs (1). PAF is a Lewy body disease with prominent loss of peripheral sympathetic noradrenergic nerves.

As isolated sympathetic AF can be the presenting feature of a central nervous system (CNS) synucleinopathy like Parkinson’s disease (PD) or multiple system atrophy (MSA), a 5-year duration of autonomic symptoms is required to make a diagnosis of PAF. Nevertheless, description of PAF patients evolving to PD (2) or MSA has been reported (3).

Hence, because the prognosis of MSA and PD is so different (4), the use of ancillary tests for predictive features in patients with PAF or isolated AF can be extremely valuable. Here, we report three cases (two with autopsy-proven diagnosis) that had isolated AF for many years before converting to MSA or PD. Clinical assessment and diagnostic work-up throughout their disease course were carried out at our Institution. All patients had given their written informed consent to personal data processing for research purposes.

## Background

### Case Report 1

This patient (aged 50–60 years) with unremarkable past medical and family history was admitted with a 7-year history of urinary urgency, frequency, and impaired urinary flow, associated with erectile dysfunction. The patient had previously undergone an urodynamic study consistent with neurogenic bladder. The patient also had episodes of blurred vision and instability on standing, particularly in hot weather and after meals. Neurological examination was normal. Blood pressure (BP) measurement revealed OH (5) confirmed by cardiovascular autonomic tests (6) that also showed absence of phase IV BP overshoot of the Valsalva maneuver, a sign of sympathetic failure. BP exaggerated response to intravenous infusion of noradrenaline (NA) (denervation supersensitivity) consistent with a sympathetic efferent dysfunction was also observed. A 24-h assessment of BP revealed supine systolic hypertension and absence of the physiological nocturnal decrease of BP (non-dipper pattern). A diagnosis of neurogenic OH and supine hypertension was made. Secondary causes of OH (7) were excluded (normal hematological, biochemical, immunological tests, and cardiological evaluation; negative paraneoplastic, infectious, metabolic, and autoimmune screening tests including antibodies to the neuronal nicotinic acetylcholine receptor; normal brain MRI, cerebrospinal fluid analysis, nerve conduction studies, and electromyography). Iodine-123-meta-iodobenzylguanidine (MIBG) myocardial scintigraphy showed normal cardiac sympathetic innervation (the patient did not take any drug that could possibly interfere with results). Despite this finding, considering the long duration of AF, the normal neurological examination after 7 years from autonomic symptom onset and the results of pharmacological tests, a diagnosis of possible PAF was made. The patient started treatment with droxidopa and fludrocortisone, with good response. During the following year (8 years after autonomic symptom onset), the patient experienced a worsening of autonomic symptoms and complained of less dexterity in the right leg. On examination, the patient presented with rigidity and bradykinesia of the right limbs, slight cerebellar ataxia, and bilateral hyperreflexia with Babinski sign. Brain MRI was still normal. Dopamine transporter imaging (DaTSCAN) showed reduced uptake in the caudate and putamen bilaterally. Cardiovascular autonomic tests confirmed previous results. Nocturnal videopolysomnography (VPSG) showed physiological muscle atonia in REM sleep and absence of *stridor*. Patient started treatment with levodopa without any significant motor improvement and developed orolingual dyskinesias. Due to the presence of cerebellar signs and absence of LD responsiveness, a diagnosis of probable MSA with predominant parkinsonism was made. Over the following 3 years, the patient developed hypophonia, symmetrical and severe parkinsonism with camptocormic posture and Pisa syndrome and also showed a worsening of cerebellar ataxia. The patient became wheelchair bound due to severe cerebellar ataxia and OH. The patient died of pneumonia 11 years after autonomic symptom onset.

### Case Report 2

This patient had been entirely well until the age of 46–50 years, when complained of reduced urinary stream associated with incomplete bladder emptying and sexual dysfunction. Treatment with anticholinergic drugs was ineffective. The patient started intermittent self-catheterization and suffered from recurrent urinary tract infections. Seven years later, the patient reported episodes of blurred vision and dizziness on standing and after exercise with occasional transient loss of consciousness, and constipation. By the same time, the patient’s mate reported episodes of sleep talking and acting out of dreams. The patient also presented with episodes of nocturnal awakening with breathing difficulty; a diagnosis of moderate OSAS was made by another Institution and continuous positive airway pressure therapy was introduced. Three years later, the patient was referred to our Clinic. Neurological examination was normal. Cardiovascular autonomic tests gave results consistent with neurogenic OH and failure of sympathetic and parasympathetic branches of the autonomic nervous system. Pharmacological studies with infusion of increasing doses of NA showed moderate noradrenergic receptors’ supersensitivity. Secondary causes of OH were excluded. MIBG myocardial scintigraphy was normal. Urodynamic studies showed atonic bladder with normal compliance and reduced sensitivity. Brain MRI and DaTSCAN were normal. The patient was diagnosed with PAF and started treatment with fludrocortisone with good response. The following year (11 years after autonomic symptom onset) the patient noticed difficulty in coordinating movements and speech impediment. The patient also reported loss of sweating in the whole body and cold hands. Neurological examination at this time showed mild limb and gait ataxia, hypotonia, scanning speech, horizontal nystagmus, and bilateral Babinski sign. Cardiovascular autonomic tests confirmed previous results. A 24-h assessment of BP showed a non-dipper pattern. VPSG showed absence of physiological muscle atonia in REM sleep, severe OSAS, and laryngeal *stridor*. A diagnosis of probable MSA with predominant cerebellar features was made. During the follow-up, the patient showed a progressive worsening of his general conditions. The patient died of respiratory failure during sleep 14 years after autonomic symptom onset.

### Case Report 3

This is a 65–70-year-old patient with a past medical history of hypertension and mild chronic kidney failure, who developed in the previous year dizziness and light-headedness after standing, worse after meals, in hot climate, and after mild exercise. The patient had suffered for the past 5 years from urinary urgency, frequency, and sexual dysfunction. Neurological examination was normal. Cardiovascular autonomic tests and 24-h BP monitoring confirmed neurogenic OH and cardiovascular AF. Secondary causes of OH were excluded. Brain MRI and DaTSCAN were normal. MIBG myocardial scintigraphy was consistent with sympathetic cardiac denervation. VPSG showed presence of physiological muscle atonia in REM sleep and absence of respiratory disturbances. The patient received the diagnosis of PAF and started treatment with midodrine. The patient was assessed regularly at our Institution and remained stable during follow-up. The patient died of acute myocardial infarction 6 years later (9 years after autonomic symptom onset).

### Pathology

At autopsy, the left cortical and cerebellar hemispheres and the left half of the brainstem were immersed in 10% buffered formalin, whereas parts of the right brain were frozen and stored at −80°C. In addition to routine histochemical stainings, monoclonal antibodies specific for the proteins beta-amyloid (clone 4G8), phospho-tau (clone AT8), and alpha-synuclein (clone LB509) were employed for immunohistochemical studies.

#### Case 1

Macroscopic examination revealed a diffuse tissue softening, especially involving the cerebral cortex, as well as a moderate atrophy of the striatum, brainstem, and cerebellum. Microscopic examination showed a moderate, focally severe, neuronal loss, and gliosis involving the substantia nigra, putamen and caudate nuclei, dentate nucleus of cerebellum, and cerebellar Purkinje cells. Additional findings included a moderate to severe demyelination and gliosis of cerebellar white matter, the presence of numerous “red neurons” in the cerebral cortex and hippocampus and petechial hemorrhages associated with microglial infiltration and astrogliosis involving the periventricular areas of the hypothalamus, thalamus, and mesencephalus.

**Figure 1:**
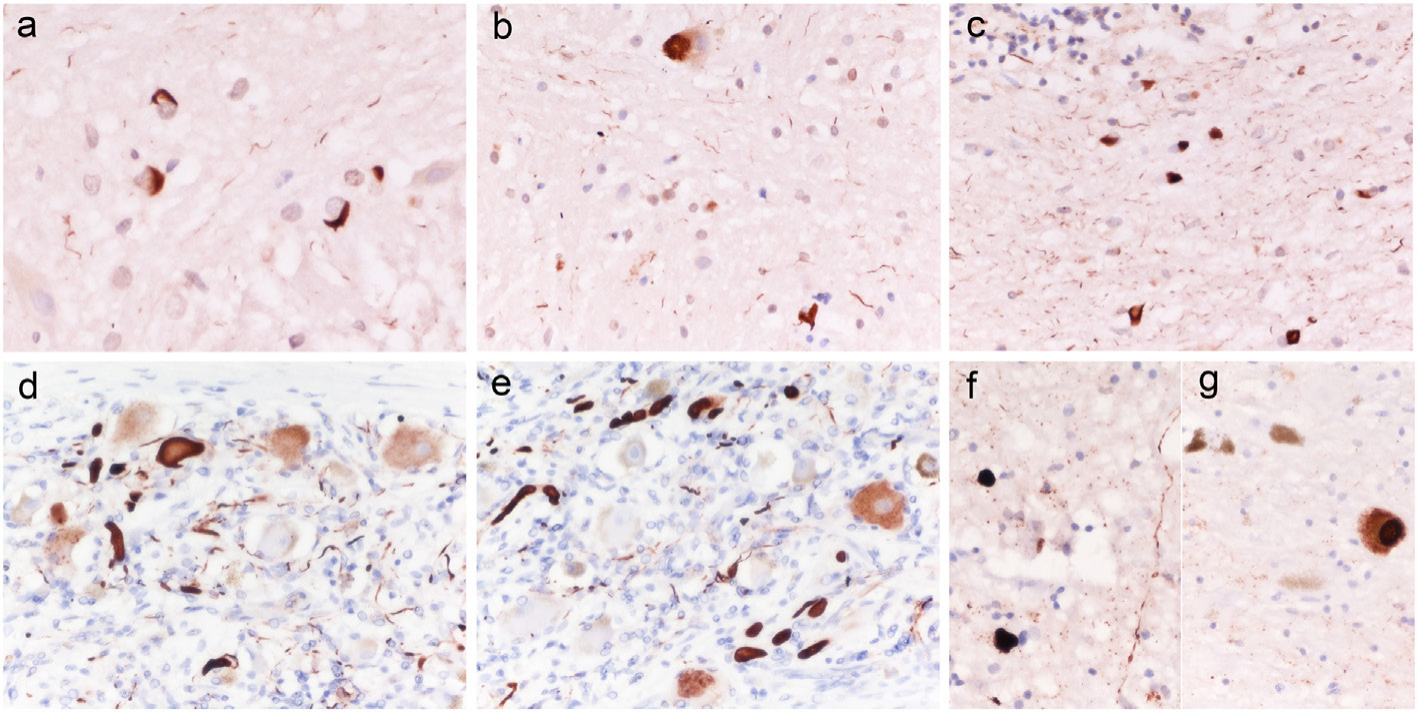
**Alpha-synuclein histopathology in patients 1 and 3**. Case 1: alpha-synuclein aggregates consistent with glial cytoplasmic inclusions (GCI) in the white matter of basis pons [**(A)** ×400]; isolated neuronal cytoplasm inclusions and GCI in the basis pons [**(B)** ×400], numerous GCI in the white matter of cerebellum [**(C)** ×400]; Case 3: Lewy body like intraneuronal and neuritic aggregates of alpha-synuclein in peripheral sympathetic ganglia **(D,E)**, medulla oblongata **(F)**, and substantia nigra **(G)**. Original magnification [**(D)** ×400; **(E,F)** ×200].

Immunohistochemistry revealed numerous intracellular protein aggregates of alpha-synuclein in oligodendroglial cells, consistent with glial cytoplasmic inclusions, in the white matter of basis pons (Figure 1A), cerebellum (Figure 1B), mesencephalon, medulla oblongata, and to a lesser extent in the thalamus and basal ganglia. Neuronal cytoplasm inclusions and neurite inclusions were sparse and limited to the basilar pontine nuclei (Figure 1C).

Neuropathological diagnosis was MSA associated with hypoxic/ischemic changes and Wernicke encephalopathy.

#### Case 3

Macroscopic examination was unremarkable except for a mild diffuse cerebral cortical atrophy. Microscopic examination of H&E stained sections only showed sparse amyloid plaques in the cerebral cortex. Immunostaining for alpha-synuclein revealed abnormal intracellular aggregates of the protein, consistent with Lewy or Lewy-like bodies and neuritis, in the peripheral sympathetic ganglia (Figures 1D,E), in the dorsal vagal nucleus (Figure 1F), locus coeruleus, brainstem reticular formation, and substantia nigra (Figure 1G) (Braak stage III). Quantitatively, while the abnormal protein aggregates were numerous in the peripheral ganglion, only rare sparse deposits were seen in the CNS structures. A-beta staining demonstrated positive diffuse and core-centered plaques in the cerebral cortex of all lobes, and multifocal deposits in the striatum, amygdala (Thal phase III), whereas P-Tau immunostaining was negative. The score for level of Alzheimer’s disease (AD) neuropathological change according to NIA-AA criteria (8) was A2 B0.

Neuropathological diagnosis was Lewy body disease involving both the peripheral nervous system (e.g., autonomic ganglia) and CNS (Braak stage III) associated with mild AD neuropathological changes.

## Discussion

We report two cases (one autopsy confirmed) of long-standing isolated autonomic failure that converted to MSA after 8 and 11 years from symptom onset, respectively, and a third case diagnosed with PAF that had autopsy findings consistent with Braak staging for PD. First, our cases confirm that even after a period of 5 years necessary to make the diagnosis, PAF patients can still evolve into a CNS synucleinopathy, consistent with previous reports (2). Second, our paper shows that MIBG myocardial scintigraphy performed in patients with isolated neurogenic OH is predictive of the conversion to MSA. MIBG myocardial scintigraphy is used to assess cardiac sympathetic innervation, as MIBG uptake in the myocardium reflects the density of postganglionic sympathetic nerve fibers. Due to the different pathophysiological mechanisms underlying AF in these two conditions (i.e., a postganglionic impairment in PD and preganglionic in MSA) (9) a reduced uptake of MIBG is observed in PD patients and a normal uptake in MSA patients (10). However, it is important to keep in mind that while PD + OH usually show abnormal MIBG uptake, PD without OH may display a normal scan (10). PAF patients, along with PD + OH patients, usually show cardiac denervation, consistent with a postganglionic sympathetic nerves involvement (10). MSA may also show a decrease in cardiac uptake of MIBG in few cases, especially with longer disease duration (11). Case 1 and 2 of our series were diagnosed with PAF because of the long duration of isolated AF and exclusion of secondary causes. However, they presented with a normal MIBG myocardial scintigraphy, which was the only exam that correctly predicted the diagnosis of MSA subsequently. Case 3 was diagnosed as PAF in life, but had neuropathological findings consistent with Braak staging at autopsy. We can speculate that if this patient had lived longer, the patient would have developed PD associated with AF. Hence, we suggest that MIBG myocardial scintigraphy, when performed in patients with isolated AF, may be a valuable predictor of conversion to MSA. On the contrary, the role of such test in patients with parkinsonism irrespective of the presence of OH is still to be clarified. The strengths of our study were the accurate clinical assessment of each patient performed at our Institution by movement disorder expert neurologists, combined with instrumental tests for autonomic function and sleep, the long follow-up, the autopsy-proven diagnosis in two cases and the high probability level of diagnosis of the third case, based on current consensus criteria and absence of features suggesting other diagnosis. The number of our cases does not allow firm conclusions; therefore, prospective studies with large cohorts of patients are needed to confirm our results. However, our findings may still be valuable to direct future research in this interesting field.

## Ethics Statement

The authors confirm that the approval of an institutional review board was not required for this work. All subjects gave written informed consent in accordance with the Declaration of Helsinki.

## Author Contributions

FB: data acquisition, drafting of manuscript, critical revisions, and final approval. GC-B: data acquisition, drafting of manuscript, critical revisions, and final approval. AC: test execution, critical revisions, and final approval. GB: test execution, critical revisions, and final approval. MC: test execution, critical revisions, and final approval. PP: pathology, critical revisions, and final approval. PC: conceptualization of study design, critical revisions, and final approval.

## Conflict of Interest Statement

PC had consulting fee or honorarium and support for travel to meetings from Abbvie, Allergan, Chiesi, Lilly, Teva, UCB, and Zambon. The other authors declare no conflicts of interest.

## References

[1] The consensus committee of the American Autonomic Society and the American Academy of Neurology. Consensus statement on the definition of orthostatic hypotension, pure autonomic failure, and multiple system atrophy. Neurology (1996) 46:1470 10.1212/WNL.46.5.1470 8628505

[2] Kaufmann H Nahm K Purohit D Wolfe D. Autonomic failure as the initial presentation of Parkinson disease and dementia with Lewy bodies. Neurology (2004) 63:1093–5. 10.1212/01.WNL.0000138500.73671.DC 15452307

[3] Iodice V Lipp A Ahlskog JE Sandroni P Fealey RD Parisi JE Autopsy confirmed multiple system atrophy cases: mayo experience and role of autonomic function tests. J Neurol Neurosurg Psychiatry (2012) 83:453–9. 10.1136/jnnp-2011-301068 22228725 PMC3454474

[4] Goldstein DS Holmes C Sharabi Y Wu T Survival in synucleinopathies. A prospective cohort study. Neurology (2015) 85:1554–61. 10.1212/WNL.0000000000002086 26432848 PMC4642141

[5] Freeman R Wieling W Axelrod FB Benditt DG Benarroch E Biaggioni I Consensus statement on the definition of orthostatic hypotension, neurally mediated syncope and the postural tachycardia syndrome. Clin Auton Res (2011) 21:69–72. 10.1007/s10286-011-0119-5 21431947

[6] Baschieri F Calandra-Buonaura G Doria A Mastrolilli F Palareti A Barletta G Cardiovascular autonomic testing performed with a new integrated instrumental approach is useful in differentiating MSA-P from PD at an early stage. Parkinsonism Relat Disord (2015) 21:477–82. 10.1016/j.parkreldis.2015.02.011 25749354

[7] Goldstein DS Sharabi Y Neurogenic orthostatic hypotension: a pathophysiological approach. Circulation (2009) 119:139–41. 10.1161/CIRCULATIONAHA.108.805887 19124673 PMC4182314

[8] Montine TJ Phelps CH Beach TG Bigio EH Cairns NJ Dickson DW National institute on aging-Alzheimer’s association guidelines for the neuropathologic assessment of Alzheimer’s disease: a practical approach. Acta Neuropathol (2012) 123:1–11. 10.1007/s00401-011-0910-3 22101365 PMC3268003

[9] Iodice V Low DA Vichayanrat E Mathias CJ. Cardiovascular autonomic dysfunction in MSA and Parkinson’s disease: similarities and differences. J Neurol Sci (2011) 310:133–8. 10.1016/j.jns.2011.07.014 21849175

[10] Goldstein DS Holmes C Li ST Bruce S Verhagen Metman L Cannon RO Cardiac sympathetic denervation in Parkinson disease. Ann Intern Med (2000) 133:338–47. 10.7326/0003-4819-133-5-200009050-00009 10979878

[11] Orimo S Kanazawa T Nakamura A Uchihara T Mori F Kakita A Degeneration of cardiac sympathetic nerve can occur in multiple system atrophy. Acta Neuropathol (2007) 113:81–6. 10.1007/s00401-006-0160-y 17089131

